# Supporting pollinators in urban gardens: floral richness and abundance influence flower visitor interactions regardless of the surrounding landscape

**DOI:** 10.1007/s11252-025-01848-7

**Published:** 2025-11-14

**Authors:** Emma Plant, Ria Dunkley, Davide M. Dominoni, Dominic J. McCafferty

**Affiliations:** 1 School of Biodiversity, One Health, and Veterinary Medicine, College of Medical, Veterinary and Life Sciences, University of Glasgow, Graham Kerr Building, Glasgow, G12 8QQ Scotland; 2 School of Education, College of Social Sciences, University of Glasgow, St Andrew’s Building, G3 6NH Glasgow, Scotland; 3 Scottish Centre for Ecology and the Natural Environment, School of Biodiversity, One Health and Veterinary Medicine, College of Medical, Veterinary and Life Sciences, University of Glasgow, Glasgow, G63 0AW Scotland

**Keywords:** Plant-flower visitor interactions, Non-native plants, Private gardens, Local and landscape variables

## Abstract

Private gardens contribute to a large area of land within cities. Yet, little is known about the value of private gardens in pollinator conservation in terms of their management and position within the urban matrix. We investigated seasonal patterns in how environmental variables influenced plant-flower visitor interactions and the network structure in private gardens. We collected plant-flower visitor data in 18 private gardens, across Glasgow, Scotland, simultaneously recording floral abundance and richness, plant origin, garden size, land use diversity and the area of green space surrounding gardens. We observed a decline in native plant abundance from June until September, but flower visits to native and non-native plants did not track this seasonal pattern. Flower visits to native and non-native plants differed depending on whether visits were analysed at the community or plant species level. Furthermore, increasing non-native floral abundance showed a greater increase in flower visitors compared to when native floral abundance increased. Plant-flower visitor networks varied seasonally, becoming more specialised in June and September, but were not influenced by other environmental variables. Overall, higher floral richness and abundance within a garden influenced flower visits, independent of the surrounding landscape. Our results indicate that private gardens can support pollinators in cities, regardless of the urban context in which they are situated and that pollinator diversity may be improved in gardens by increasing floral diversity. We provide a list of attractive plants species to help inform floral plantings in gardens.

**Supplementary information:**

The online version contains supplementary material available at 10.1007/s11252-025-01848-7.

## Introduction

Pollinators provide an important ecosystem service, in that they facilitate the reproduction of flowering plants (Ollerton et al. 2011), including 76% of global crop species (Klein et al. 2007). Thus, pollinator declines because of climate and land use change, are concerning. The effects of urbanisation, which sees the conversion of natural landscapes to those dominated by impervious surfaces, on pollinator diversity are therefore of increasing interest due to the potential conservation value of urban green spaces. Urban environments support fewer pollinator species and a more homogenised community than natural areas due to environmental pressures that select on pollinator traits such as body size and diet breadth (Ayers and Rehan 2021; Wenzel et al. 2020). For example, the prevalence of impervious surfaces in urban areas limits nesting availability for ground-nesting bees (Geslin et al. 2016). Yet, mechanisms of selection against certain pollinator traits are more nuanced, as pollinators possess multiple traits which are influenced by multiple interacting environmental drivers acting at both local and landscape scales (Bennett and Lovell 2019). However, compared to agricultural areas, urban areas can be strongholds for certain pollinator species due to the comparatively heterogeneous landscape and higher floral richness in cities (Theodorou, 2017).

Urban green spaces vary in their habitat suitability for pollinators (Baldock et al. 2019; Daniels et al. 2020; Dylewski et al. 2019). Few comprehensive studies of urban green spaces have been conducted, yet private, residential gardens have been cited as one of the best habitats for pollinators within the urban matrix (Baldock et al. 2019). Despite being small, collectively private gardens contribute to a large area of pollinator habitat (Baldock et al. 2019). Gardens often have high floral abundances, yet many of the floral species in gardens are non-native (Erickson et al. 2020), which are often unsuitable for specialist pollinator species (Chrobock et al. 2013; Salisbury et al. 2015). Nonetheless, some non-native plant species are highly attractive to pollinators (Salisbury et al. 2015). Many studies have investigated how the characteristics of urban green space habitats influence pollinator diversity (Baldock et al. 2019; Daniels et al. 2020; Dylewski et al. 2019; Tassin de Montaigu and Goulson, 2024), but thus far, there has been comparatively little focus on plant-pollinator networks in private green spaces.

Plant-pollinator networks are mutualistic interaction networks which form the basis of biodiversity preservation as their structure relates to their stability and functioning (Elle et al. 2012; Ferreira et al. 2013). Thus, studying interaction networks can hold valuable information for conservation (Elle et al. 2012). Within gardens, the local habitat varies significantly, with differences in their floral diversity, composition and garden size; all of which may influence plant-pollinator networks. For example, increased floral richness allows niche segregation within pollinator species by reducing competition for floral resources (Gómez-Martínez et al. 2022). Consequently, specialisation increases with floral richness. Recently, studies have focused on the roles of non-native plants in structuring plant-pollinator networks (Staab et al. 2020; Zaninotto et al. 2023). Pollinators show preferences for native plants (Chrobock et al. 2013; Lowenstein et al. 2019; Salisbury et al. 2015), and many specialist pollinators are unable to forage on non-native plants, leading to a loss of specialists from the environment (Seitz et al. 2020). However, some studies have shown that certain non-native plants are attractive to pollinators later in the flowering season when native plants are scarce (Staab et al. 2020; Zaninotto et al. 2023). These late-season non-native plants either substitute for native plants, having little effect on the network (Staab et al. 2020) or decrease network specialisation by attracting generalist pollinators (Zaninotto et al. 2023). Finally, garden size may influence network structure as smaller, specialist pollinators rely on larger patches in fragmented habitats (Bommarco et al. 2010). As size restricts foraging range, and specialism restricts pollinator diet, smaller gardens may result in fewer specialist pollinators (Bommarco et al. 2010; Herrmann et al. 2023).

Additional to the local characteristics of gardens, the context in which a green space is situated within the urban matrix may influence plant-pollinator networks (Herrmann et al. 2023). Studies on urban-rural gradients have found urbanisation to positively influence network specialisation (Baldock et al. 2015; Proesmans et al. 2024; Theodorou et al. 2017). Moreover, grasslands surrounded by more impervious surfaces have higher species specialisation (Herrmann et al. 2023). However, these studies suggest that increased specialisation is due to abundant non-native plant species rather than a consequence of the impervious surface around a site. Furthermore, how the surrounding urban matrix is categorised may affect the results on plant-pollinator networks. For example, land use diversity positively influenced network specialisation and nestedness (the extent to which specialists interact with a subset of species that generalists also interact with), whereas the abundance of green space had no effect (Graf et al. 2024). Here, land use diversity is thought to increase both nesting and floral resources surrounding a site (Graf et al. 2024). Overall, the effect of the surrounding landscape on plant-pollinator networks remains unclear.

Studies on plant-pollinator networks in urban green spaces often consider local and landscape variables or plant origin in isolation, even though these environmental drivers likely interact to influence plant-pollinator networks. In this study, we therefore examined which environmental variables (floral abundance, richness, plant origin, garden size and surrounding landscape) of private gardens influenced pollinator interactions across the season. The aims and hypotheses tested in this study are as follows: (1) To assess the effect of garden and landscape level environmental drivers, including monthly variation, on flower visitation and community similarity. Increasing floral abundance and richness of native plants will increase flower visits (H1). Native plants will receive more flower visits than non-native plants when tested at the plant community and species level (H2). Flower visitor community composition will differ between gardens with different floral abundance and richness, and across months (H3). (2) Determine the influence of garden and landscape characteristics on plant-pollinator network structure, including monthly variation. Network specialisation will increase with floral richness due to niche partitioning (H4). Networks will become less specialised, more nested and with higher interaction evenness in September compared to June due to increased abundances of non-native plants (H5). (3) Examine whether patterns in flower visits and network structure are driven by a single dominant species. Environmental variables will still significantly affect network structure and flower visitor interactions when the most dominant species is excluded (H6). Finally, to promote pollinator-friendly gardening and guide floral plantings, we identified plant species preferred by pollinators using null networks.

## Methods

A total of 18 gardens were sampled across the City of Glasgow, Scotland, extending to a 5 km buffer from the city boundary (online resource 1, Figure S.1), with individual gardens sampled on separate days once a month from 1/6/23–30/9/23. Due to the difficulty in recruiting garden owners, spring was not sampled in this study. Gardens were recruited via social media advertisement and selected on a first-come, first-selected basis, provided they were located over 1 km away from other chosen gardens. While this method of recruitment may have introduced a selection bias of sample sites towards pollinator friendly management in gardens, the gardens were visited before sampling commenced to ensure a range of management styles was included in the study. This resulted in a range of mean floral abundances (15.38 ± 13.69 to 159.38 ± 222.48) and richness (1.38 ± 1.51 to 6 ± 5.22) across the four study months present at sites. A minimum of 1 km was left between sites, as this is greater than the average foraging range of most flower visitors (Kendall et al. 2022), thus ensuring sites were independent. However, some studies have found maximum foraging ranges to be greater than 1 km (Kendall et al. 2022), thus we tested for spatial autocorrelation during data analysis (see below).

### *Floral resources*

At the start of each sampling day, floral resources were inventoried. Transects (0.25 m width), were used to record a floral inventory. Due to differences in the shape and size of gardens, transects were made equal to the length of the garden and were stratified to use one transect per 5 m width of each vegetation type (grassy areas, floral borders and vegetable patches). Transects lengths ranged from 10 m to 60 m (mean ± SD: 25.44 ± 14.60). Plants that fell within the transect were identified to genus or species, and the number of floral units (individual flowers) on each plant was counted. Apiaceae umbels and Asteraceae flower heads were counted as one floral unit. Shrubs and trees (up to a height of 1.5 m) were included in the count, with the number of flowers on one branch counted and multiplied by an estimate of the number of branches within the transect. One observer, (E.P), carried out the plant recording and identifying using botanical keys (Rose and O’Reilly 2006). Garden owners would provide information where possible on the identification of non-native ornamental species which would be corroborated using pictures of the plants taken in the field and online resources. Where possible plants were identified to the lowest possible taxonomic level following the International Code of Nomenclature for algae, fungi and plants. Plant origin was classified using the BSBI atlas (Stroh et al. 2023), in which the GB status of the plant was used, and neophytes and aliens were grouped as non-native and archetypes as native. Two plant species were not categorised into these groups by the BSBI atlas and were classified as unknown.

### *Plant-flower visitor network*

After creating floral inventories, flower visitors were identified and counted. Flower visitor sampling would be carried out between 09.30 and 11.30 (morning) and 12.30 and 17.00 (afternoon), provided the temperature was above 10 degrees, on clear days with wind speed below 15 km/h. One flower of each species was randomly chosen to be observed for its flower visitor interactions and randomly assigned to morning or afternoon recording. Flowers were observed by a single observer (E.P.), for 20 min after a 2-minute settling period in which the observer had positioned themselves nearby the flower. Any Syrphidae, Papilionoidea or Apidae observed to touch the reproductive part of the flower were collected for identification. Any flower visitors that were not identifiable in the field were euthanised using ethyl acetate vapours or by freezing within 30 min of collection. Flower visitors were identified by the lead author using the Field Guide to Bees of Great Britain and Ireland (Falk and Lewington 2018) and British Hoverflies (Stubbs and Falk 2002). We recognise these observations only consider flower visits, meaning actual pollination may not have occurred in these flower visits. The maximum number of plant species observed in one day, in on garden, was 17 (mean ± SD = 4.09 ± 2.40). As sampling in an individual garden was limited to one day a month, plant observations were unable to be longer than 20 min each, with 340 min being the maximum total observation time in one garden. Finally, sampling events were separated by at least three weeks to ensure insect populations recovered and to maintain temporal independence of sampling events.

Quantitative plant-flower visitor networks were built using the Bipartite package in R (Dormann et al. 2008). We removed networks with fewer than two species per level to calculate network-level indices, as this is the smallest possible network size that allows network indices to be calculated. For species indices, flower visitors observed once in the sampling period were removed. For each network (per site, per month), we calculated the following indices:

- H_2_’-The specialisation of the whole network describes the level of selectivity within the network. This network ranges from 0 (no specialisation) to 1 (complete specialisation) (Blüthgen et al. 2008).
- Interaction evenness- The spread of interactions within the network. Higher interaction evenness equates to an even spread of interactions throughout the network. Ranging from 0 to 1, a network with fewer interactions that occur in high frequency will have lower interaction evenness (Blüthgen et al. 2008).
- Weighted Niche Overlap based on Decreasing Fill (WNODF)- a measure of nestedness, the extent to which specialists interact with a subset of species, which generalists also interact with (Blüthgen et al. 2008).
- d'- Species specialisation, the level of selectivity of a species, ranging from 0 to 1(Blüthgen et al. 2006).
- Network size: the number of species in each level of the network (number of plant species plus the number of flower visitor species). This was calculated to use as a control when modelling network indices, as quantitative network metrics are sensitive to network size.

### *Environmental variables*

The total area of the garden was measured in m^2^ using QGIS v3.32.1 (Herrmann et al. 2023; Stewart et al. 2018). The Urban Atlas data set from Copernicus (European Union 2020) was used to describe land use types surrounding sites (resolution 0.25 ha). We quantified the total area of sports and leisure facilities, pastures, permanent crops, artificial non-agricultural vegetated areas, shrubs and herbaceous vegetation, arable land, forest and herbaceous vegetation association within buffers of 100 m, 200 m, 400 m, 600 m, 800 m and 1000 m to measure surrounding green space. We used the area of each land use category within the same buffers and the Shannon-Weiner index to calculate land use diversity around gardens. While these green space types may vary in their quality as flower visitor habitat in terms of their floral resources, there are no data that quantifies the quality of these spaces in Glasgow.

### Data analysis

All analysis was carried out in R version 4.4.2. All continuous explanatory variables were standardised using R’s scale function, which centres variables by subtracting their mean and dividing by the standard deviation. In all models described below, the significance of variables was tested using a backwards stepwise model selection and likelihood ratio tests (Zuur et al. 2009). Models were checked for uniformity and homoscedasticity of residuals using the DHARMa package (Hartig 2024). Collinearity, zero inflation and overdispersion of models were checked using the performance package (Lüdecke et al. 2021). We tested for spatial autocorrelation by computing Moran’s I values on the residuals of each model using the DHARMa package. No spatial autocorrelation was detected in any of the models. Finally, we fitted models for each of our response variables described below with fixed explanatory landscape variables (land use diversity and area of green space), at different landscape scales (100 m, 200 m, 400 m, 600 m, 800 m and 1000 m). We then compared model AIC values (Bozdogan 1987) to test the best landscape scale to use in models. For flower visitor richness at the community level the 400 m buffer had the lowest AIC (AIC = 520.00). In contrast, models for the other response variables did not show differences in AIC scores when comparing landscape scales, therefore 400 m was chosen as the landscape scale to be included in all models. All *p* values reported in the results section were adjusted using the false discovery rate (FDR) method.

### *Floral resources*

Differences in floral abundance and richness were tested using Generalised Linear Mixed Models (GLMMs). Floral abundance was modelled using a negative binomial distribution in the R package glmmTMB (Brooks et al. 2017) and floral richness using a Poisson distribution in the R package lme4 (Bates et al. 2015). Fixed explanatory variables included land use diversity, area of green space, month as a linear and quadratic effect, and plant origin. Garden size was included to control for larger gardens having higher floral abundances. Site was included in models as a random effect.

### *Flower visitor interactions*

Firstly, we assessed how predictor variables influenced flower visitor interactions in the whole community using GLMMs with a negative binomial distribution. Here, we used the total number of interactions and the species richness of flower visitors to all native or non-native plants as response variables. We used the continuous explanatory variables: floral richness, garden size, area of green space, land use diversity and month as a linear and quadratic term, as well as the categorical variable plant origin as fixed effects. Interactions between month and plant origin and floral richness and between month and plant origin were also included. As floral abundance per m^2^ introduced collinearity, we separately modelled floral abundance per m^2^ and its interaction with plant origin as fixed effects. Site was included as a random effect. Due to the high number of interactions from *Bombus pascuorum (~ 25% of interactions)*, we analysed the number and richness of floral visits without *B.pascuorum*. As *B.pascuorum* is a known generalist species that is capable of foraging on a range of plants (Fisogni et al. 2022), thus we wanted to assess whether the patterns in flower visitor interactions were driven by *B.pascuorum*.

Secondly, we tested floral visits at the plant species level, in that response variables were the number and richness of floral visits per plant species. Floral visits at the plant species level were modelled using negative binomial distributions and flower visitor richness at the plant species level was modelled using Poisson distributions with a zero-inflation factor. Fixed explanatory variables included garden size, area of green space, land use diversity, month as a linear and quadratic effect, plant origin and an interaction between month and plant origin. These models also included the floral abundance of plant species as a fixed effect to control for the fact that plants with higher floral abundance are more likely to attract pollinators. Site and plant species were included as random effects.

Next, we assessed the percentage of native and non-native plant species within networks that received interactions using GLMMs with Gaussian distribution. Here, we divided the number of native or non-native plant species that received flower visits by the total number of native or non-native plant species in the network. Floral abundance per m^2^, floral richness, garden size, area of green space, land use diversity and month as a linear and quadratic effect and plant origin were included as fixed effects. Interactions between month and plant origin with floral abundance and floral richness and between month and plant origin were also included. Site was a random effect.

Finally, we assessed the similarities of flower visitor communities at different sites using Hellinger-transformed species abundances. Hellinger transformations reduce the influence of highly abundant species whilst preserving ecological distances. A PERMANOVA was used to test for the significance of environmental variables (floral richness, floral abundance per m^2^, plant origin, garden size, land use diversity, area of green space around a site and month) in influencing community differences. Here we used the Adonis2 function in the R package vegan (Oksanen et al., 2024). For visualisation, NMDS plots were made using the metaMDS function in vegan (Oksanen et al., 2024) and used Bray-Curtis dissimilarity values.

### *Network indices*

We used beta distribution GLMMs in the glmmTMB R package (Brooks et al. 2017), to test for differences in H_2_’, interaction evenness and flower visitor specialisation (d’), and Gaussian distribution GLMMs for WNODF. We also modelled pollinator specialisation using a zero-inflated beta distribution; however, this did not improve the model fit. Therefore, we used the original beta regression as this was a simpler model; a similar approach was used in Zaninotto et al. 2023. The fixed explanatory variables used were plant origin, month as both a linear and quadratic effect, floral abundance per m^2^, floral richness and their interactions as well as land use diversity and area of green space. Month was modelled separately from plant origin to avoid collinearity. We included network size as a fixed effect due to its influence on network indices. Site was included as a random effect. In addition, models for flower visitor specialisation (d’) included the abundance of individual flower visitor species as a fixed effect, and flower visitor species identity as a random effect.

Again, because *B. pascuorum* interactions were highly frequent and may shape plant-flower visitor network structure, we recalculated all network indices (H_2_’, Interaction evenness, and WNODF) when *B. pascuorum* was removed from the network. Similarly, we carried out a separate analysis of species specialisation scores (d’) without *B. pascuorum* in the data set and tested *B. pascuorum* specialisation scores when all other species had been removed from the data set.

### *Plant preferences based on floral abundance*

We used the econullnetr package (Vaughan et al. 2018) to generate null models based on floral abundance, allowing us to test whether flower visitors interact with plant species more or less frequently than expected given plant species availability. As visit frequency is expected to be influenced by the abundance of available plant species, this approach enabled us to identify specific floral preferences of individual flower visitor species. If flower visitors interacted with a plant more than expected, it is assumed that the flower visitor had preferences for that plant species. The complete list of plant preferences is included in the supplementary material (Online resource 1, Table S.4).

## Results

### Floral resources

Gardens varied in their floral abundance each month (June native mean ± SD = 124.93 ± 145.12 flowers, June non-native 19.2 ± 23.67 flowers, July native 107.88 ± 76.00 flowers, July non-native 44.53 ± 26.00 flowers, August native 45.25 ± 39.50 flowers, August non-native 44.13 ± 30.50 flowers, September native 25.14 ± 17.89 flowers, September non-native 33.00 ± 22.82 flowers). Similar variation was observed in floral richness (June native: mean ± SD = 5.86 ± 2.28 flower species, June non-native 1.70 ± 1.06 flower species, July native 6.18 ± 3.11, July non-native 2.65 ± 1.11 flower species, August native 4.94 ± 2.11 flower species, August non-native 3.31 ± 1.66 flower species, September native 4.00 ± 1.52 flower species, September non-native 3.17 ± 1.64 flower species).

**Fig. 1:**
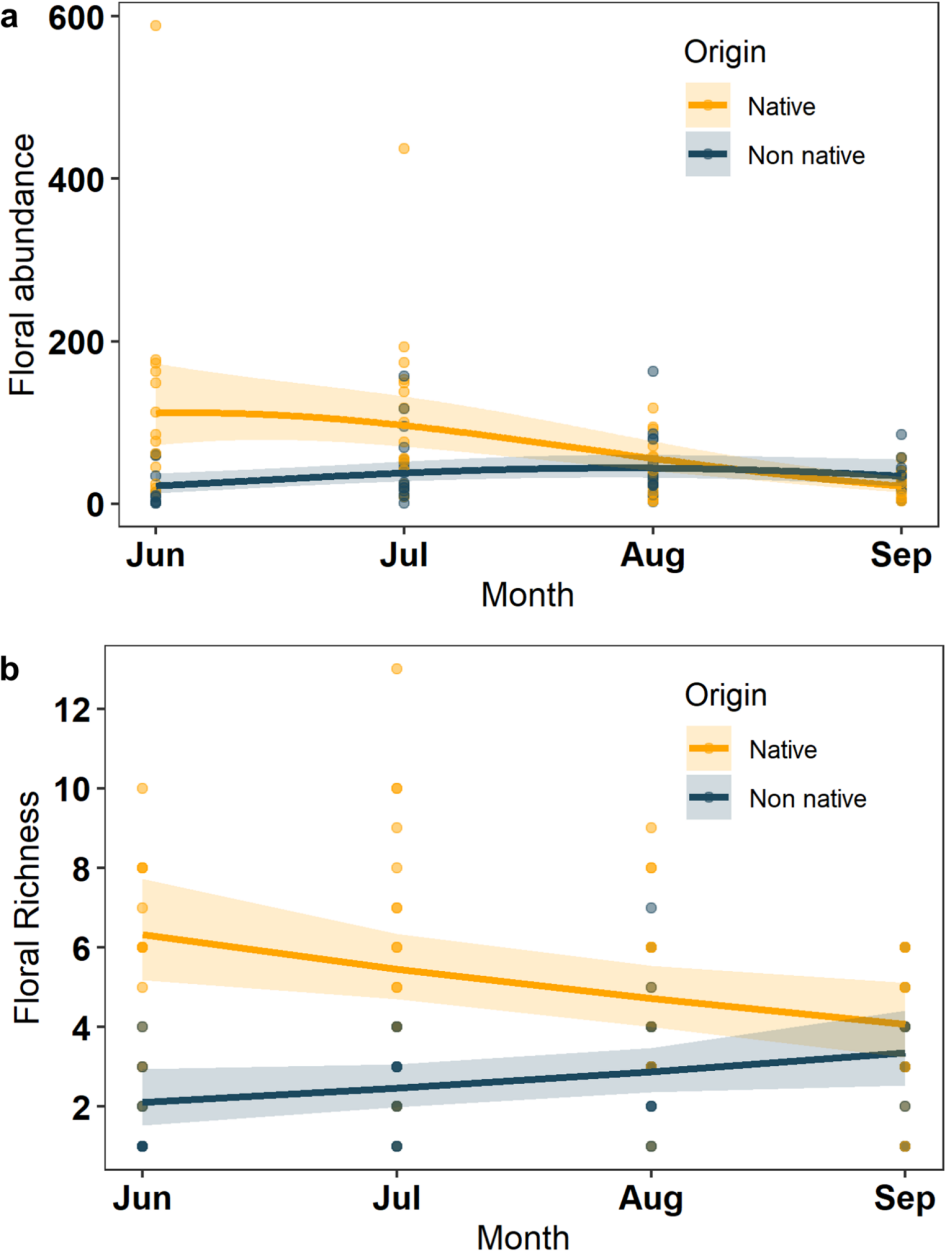
(**a**) Floral abundance and (**b**) floral richness of native and non-native plants across the four months of the study. Points show raw data for floral values collected in the field per site per month. The solid line shows the fitted regression with shaded areas representing the 95% confidence intervals

Final models for floral abundance and floral richness can be seen in Table 1. Floral abundance changed across months with higher abundances in July and August compared to June and September (Estimate=−1.08, z=−2.4, *p* = 0.024). An interaction between plant origin and month influenced floral abundance (Estimate = 0.73, z = 4.33, *p* < 0.001) (Fig. 1a). Overall, non-native plant abundance increased across the months, and native plants decreased. This pattern reflects the later phenology of non-native plants compared to native plants found in other studies (Zaninotto et al. 2023). An interaction between month and plant origin on floral richness was significant (Estimate = 0.32, z = 3.13, *p* = 0.001); again, native plant richness decreased whilst non-native plants increased across the months (Fig. 1b). Native floral richness was higher than non-native floral richness (Estimate=−0.64, z=−6.4, *p* < 0.001).

**Table 1:** *Model outputs for final models to explain response variables of flower visits (at the community and plant level, for B. pascuorum only and the percentage of plant species interacted with), floral resources (floral abundance and floral richness), network indices (H*_2_’,* interaction evenness, WNODF, d’ for all pollinators and d’ for B.pascuorum only). For each significant explanatory variable included in the final models, the table reports the estimate, standard error, z value, 95% confidence interval, and p values (adjusted using the FDR method)*

Response	Predictors	Estimate	Z	Standard error	Conf. low	Conf. high	Adj. *p* value
**Flower visits**							
Flower visits at the community level	Floral richness Origin Month Floral abundance/m2 Floral abundance*Origin	0.27 −0.30 −0.26 −0.04 0.30	3.89 −2.32 −3.60 −0.42 0.15	0.07 0.13 0.07 0.10 2.06	0.110 −0.566 −0.405 −0.25 0.008	0.403 −0.0436 −0.116 0.17 0.60	< 0.001 0.035 0.004 0.047 0.041
Flower visitor richness at the community level	Floral richness Floral abundance * Origin	0.39 0.14	7.00 1.42	0.06 0.10	0.28 −0.06	0.50 0.33	< 0.001 0.041
Flower visits at the plant level	Month Floral abundance	−0.20 0.19	−3.15 2.86	0.06 0.07	−0.33 0.06	−0.08 0.32	0.002 0.001
Flower visitor richness at the plant level	Month Origin Floral abundance	−0.16 0.33 0.16	−3.51 2.01 3.30	0.05 0.17 0.05	−0.27 0.01 0.07	−0.06 0.66 0.26	0.002 0.048 0.001
Percentage of plants to receive visits	Origin	11.25	2.45	4.59	2.14	20.4	0.027
*B.pascuorum* flower visits	Floral richness Floral abundance Origin Month*Origin Floral richness * Origin	0.02 0.31 0.12 0.48 −0.18	0.48 7.16 1.85 7.72 −3.56	0.05 0.04 0.07 0.06 0.05	−0.07 0.23 −0.008 0.36 −0.29	0.12 0.4 0.25 0.60 −0.08	0.002 < 0.001 0.027 < 0.001 < 0.001
Flower visits excluding *B.pascuorum*	Floral abundance Floral richness Month	0.35 0.47 −0.31	3.31 5.57 −3.79	0.11 0.08 0.08	0.15 0.30 −0.48	0.57 0.64 −0.15	0.001 < 0.001 < 0.001
Flower visitor richness excluding *B.pascuorum*	Floral abundance Floral richness Month Origin	0.28 0.60 −0.17 0.31	3.02 8.62 −2.74 2.16	0.09 0.07 0.06 0.15	0.10 0.47 −0.29 0.03	0.47 0.75 −0.05 0.61	0.003 < 0.001 0.013 0.036
**Floral resources**							
Floral abundance	Month Month^2 Origin Month*Origin	0.48 −1.08 −0.58 0.73	1.05 −2.40 −3.43 4.33	0.46 0.45 0.17 0.17	−0.44 −1.97 −0.91 0.40	1.38 −0.18 −0.24 1.07	< 0.001 0.024 0.027 < 0.001
Floral richness	Origin Month*Origin	−0.64 0.32	−6.40 3.13	0.1 0.10	−0.84 0.12	−0.45 0.52	< 0.001 0.001
**Network indices**							
H_2_’	Month^2	2.55	3.86	0.66	1.25	3.84	< 0.001
Interaction evenness	Month^2	−0.40	−2.64	0.15	−0.71	−0.10	0.019
WNODF	Month^2	−22.67	−2.09	10.85	−44.2	−1.16	0.038
d’ for all pollinators	Month^2	0.61	2.23	0.27	0.07	1.14	0.031
d’ for *B.pascuourm* only	Month^2	2.94	4.19	0.70	1.55	4.32	< 0.001

### Flower visitor interactions

After removing small networks, a total of 1014 flower visits were recorded in gardens, comprising 126 plant and 55 flower visitor species. *B. pascuorum* contributed to 25% of interactions (*n* = 256), followed by 17% from *Bombus lucorum agg.* (*n* = 174), and 8% from *Episyrphus balteatus* (*n* = 82). The plants that received the most visits were *Lavandula sp.* (10%, *n* = 106), *Geranium sp.* (8%, *n* = 76) and *Brassica rapa* (7%, *n* = 71). The full list of flower visitor and plant species can be found in the online resources (Online resource 1, Table S. 2 and Table S. 3, respectively).

**Fig. 2:**
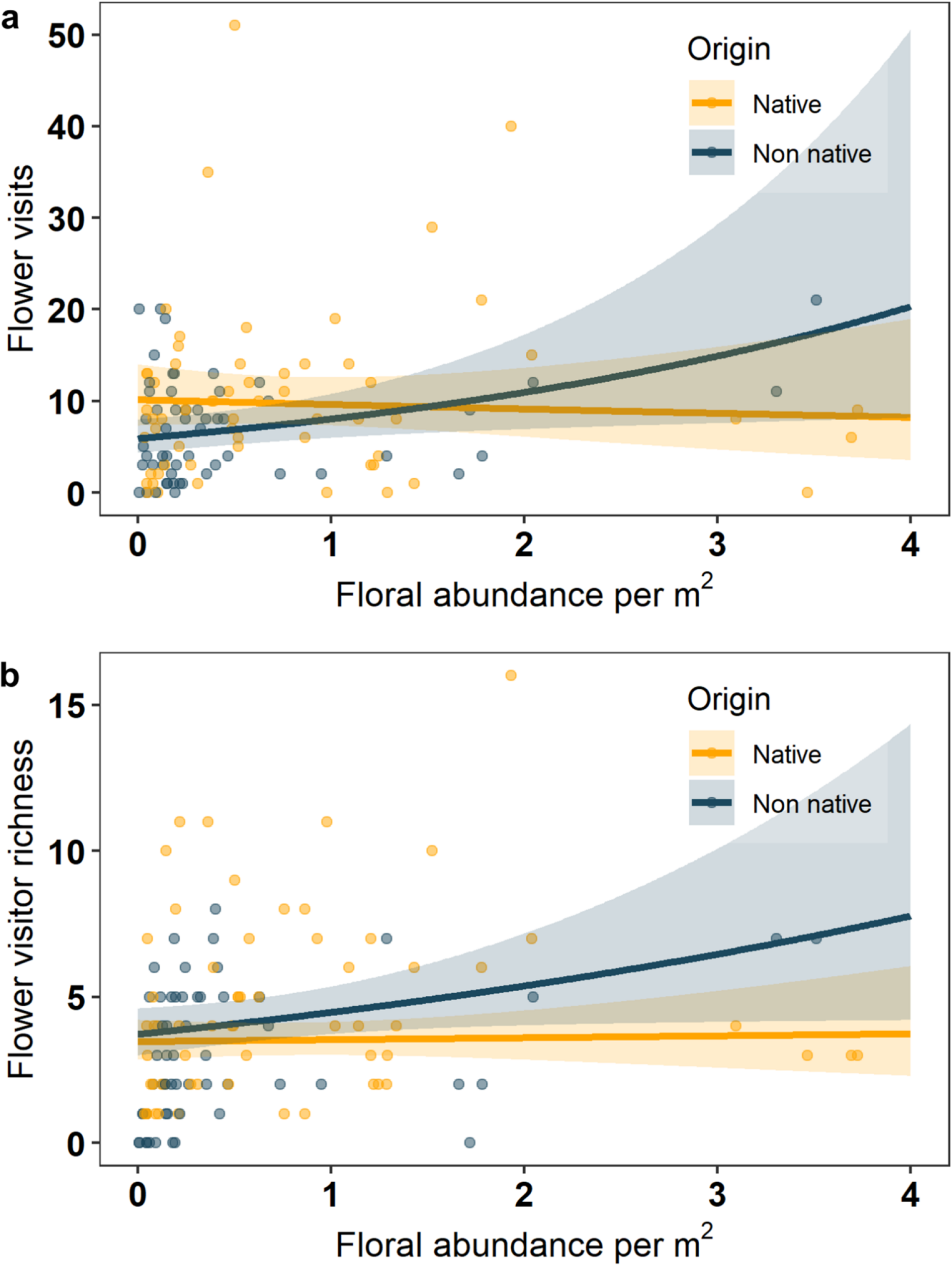
The influence of plant origin (native orange, non-native blue) on (a) the total number of flower visits at the community level as floral abundance per m^2^ increased (b) the total richness of flower visitors at the community level as floral abundance per m^2^ increased. Points show the raw values of total flower visitors to native or non-native plants per site per month. The solid line shows the fitted regression with shaded area representing the 95% confidence interval

Final models for flower visits and flower visitor richness at the community level can be seen in Table 1. The total number of flower visits was influenced by floral abundance per m^2^ depending on plant origin (floral abundance*plant origin: Estimate = 0.30, z = 0.15 *p* = 0.041; Fig. 2a). An increase in the abundance of non-native plants led to a greater increase in the number of floral visits than when native plant abundance increased. The number of flower visits was positively influenced by floral richness (Estimate = 0.27, z = 3.89, *p* < 0.001), and floral abundance per m^2^ (Estimate=−0.04, z=−0.42, *p* = 0.047). Native plants received more flower visits than non-native plants at the community level (Estimate=−0.30, z=−2.32, *p* = 0.035). Floral visits decreased from June to September (Estimate=−0.26, z=−3.60, *p* = 0.004). Notably, the richness of floral visitors was influenced by floral abundance, depending on plant origin (Estimate = 0.14, z = 1.42, *p* = 0.041) (Fig. 2b). Here, the positive relationship between flower visitor richness and plant abundance was greater for non-native plants than native plants. Furthermore, flower visitor richness increased with floral richness (Estimate = 0.39, Z = 7.00, *p* < 0.001). Neither Land use diversity nor the area of green space affected flower visitor abundance or richness.

**Fig. 3:**
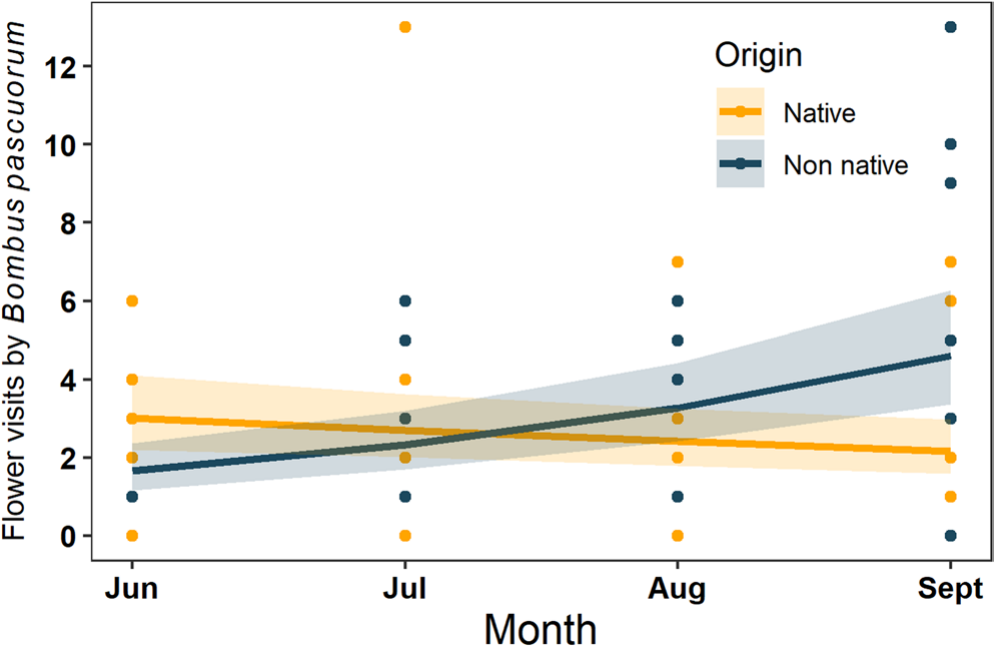
Flower visits by Bombus pascuorum, to native (orange) and non-native (blue) plants at the plant community level. Visits to native plants by *B. pascuorum* decreased from June to September, while visits to non-native plants increased. Points show the raw values of* B. pascuorum* visits to native and non-native plants in gardens per month. Lines show final model predictions of *B. pascuorum* visits and shaded areas represent the 95% confident intervals

When removing *B. pascuorum* from flower visitor interactions, different trends were observed compared to data analysed at the whole community level. Flower visits and flower visitor richness when analysed without *B. pascuorum* were no longer influence by floral abundance per m^2^ depending on plant origin. Here, flower visits increased with floral abundance per m^2^ and richness (Estimate = 0.35, z = 3.31, *p* = 0.001 and Estimate = 0.47, z = 5.57, *p* < 0.001 respectively), similar to their effects on flower visitor richness (Estimate = 0.28, z = 3.02, *p* = 0.003 and Estimate = 0.60, z = 8.62, *p* < 0.001 respectively). Both flower visits and flower visitor richness declined across the four study months when *B. pascuorum* was excluded (Estimate=−0.31, z=−3.79, *p* < 0.001 and Estimate=−0.17, z=−2.74, *p* = 0.013). When analysing *B. pascuorum* visits only, results showed a strong interaction between plant origin and month (Estimate = 0.48, z = 7.72, *p* < 0.001) (Fig. 3). Moreover, the flower visits of *B. pascuorum* increased with floral richness depending on plant origin (Estimate=−0.18, z=−3.56, *p* < 0.001), where native floral richness increased flower visits more than when non-native floral richness increased. Thus, the most dominant flower visitor species appears to influence patterns of flower visitation when the whole flower visitor community was analysed.

Final models for flower visits and flower visitor richness at the plant species level can be seen in Table 1. At the plant species level, flower visits to plants decreased from June to September (Estimate=−0.20, z=−3.15, *p* = 0.002). Moreover, flower visits to individual plants increased with the floral abundance of plant species that were visited (Estimate = 0.19, z = 2.86, *p* = 0.001). The richness of floral visitors to plant species was influenced by month (Estimate=−0.16, z=−3.51, *p* = 0.002) and plant origin (Estimate = 0.33, z = 2.01, *p* = 0.048). Non-native plants received more visitor species and the species richness of floral visitors to plant species declined from June to September, regardless of plant origin. Finally, flower visitor richness to individual flowers increased with the floral abundance of plants species that were visited (Estimate = 0.16, z = 3.30, *p* = 0.001).

Plant origin was the only determinant of the percentage of plant species that flower visitors interacted with; a higher proportion of non-native plant species received interactions, compared to native plants (Estimate = 11.25, t = 2.45, *p* = 0.027). Month did not affect the proportion of native or non-native plants within networks that received interactions.

**Fig. 4:**
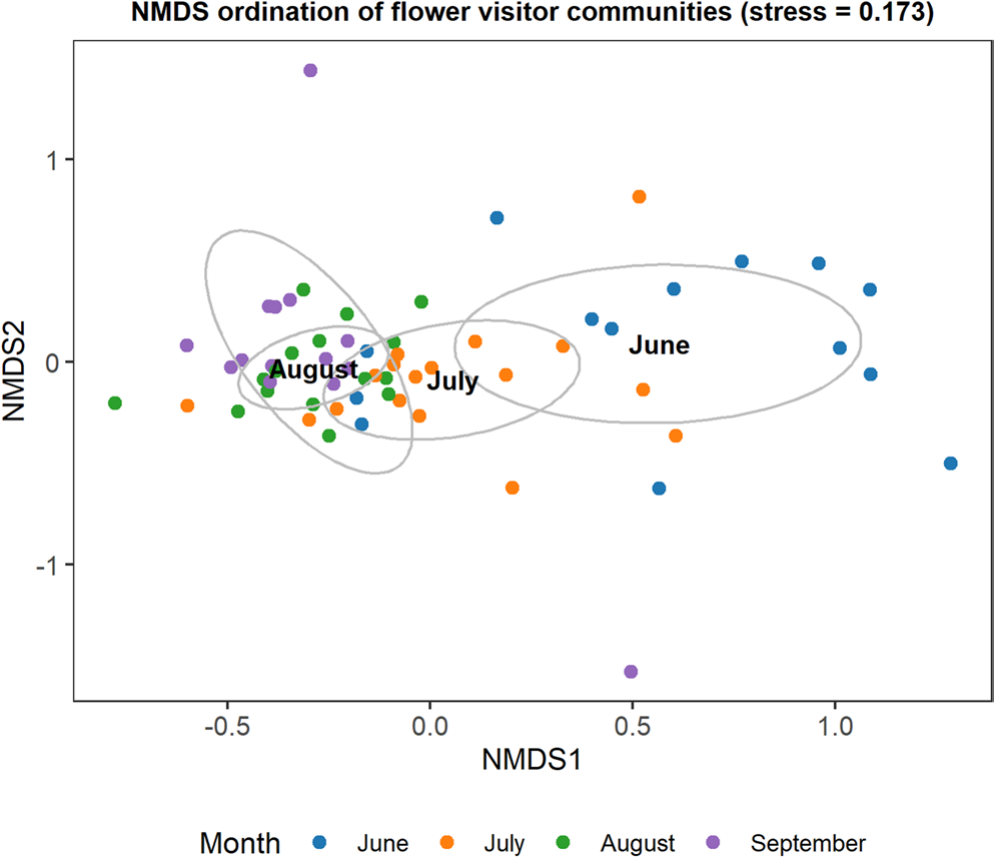
NMDS ordination plot of flower visitor communities based on Hellinger transformed species abundances and Bray-Curtis dissimilarities (stress = 0.17). Points represent sites coloured by month. 95% confidence ellipses indicate monthly groupings of sites, with the centroid labelled accordingly. Month was a significant factor in structuring flower visitor community composition. NMDS axes represent the relative distance between communities at each site, in each month

Flower visitor communities were significantly different between months (F = 2.923, DF = 3, R^2^ = 0.127, *p* = 0.001) (Fig. 4) and differed with the floral richness at the site (F = 2.137, DF = 1, R^2^ = 0.028, *p* = 0.044).

### Plant-flower visitor network structure

Network sizes ranged from 4 to 25. All networks showed a mean ± SD H_2_’ value of 0.49 ± 0.32, a mean WNODF value of 31.17 ± 22.21, and a mean interaction evenness of 0.63 ± 0.08.

**Fig. 5:**
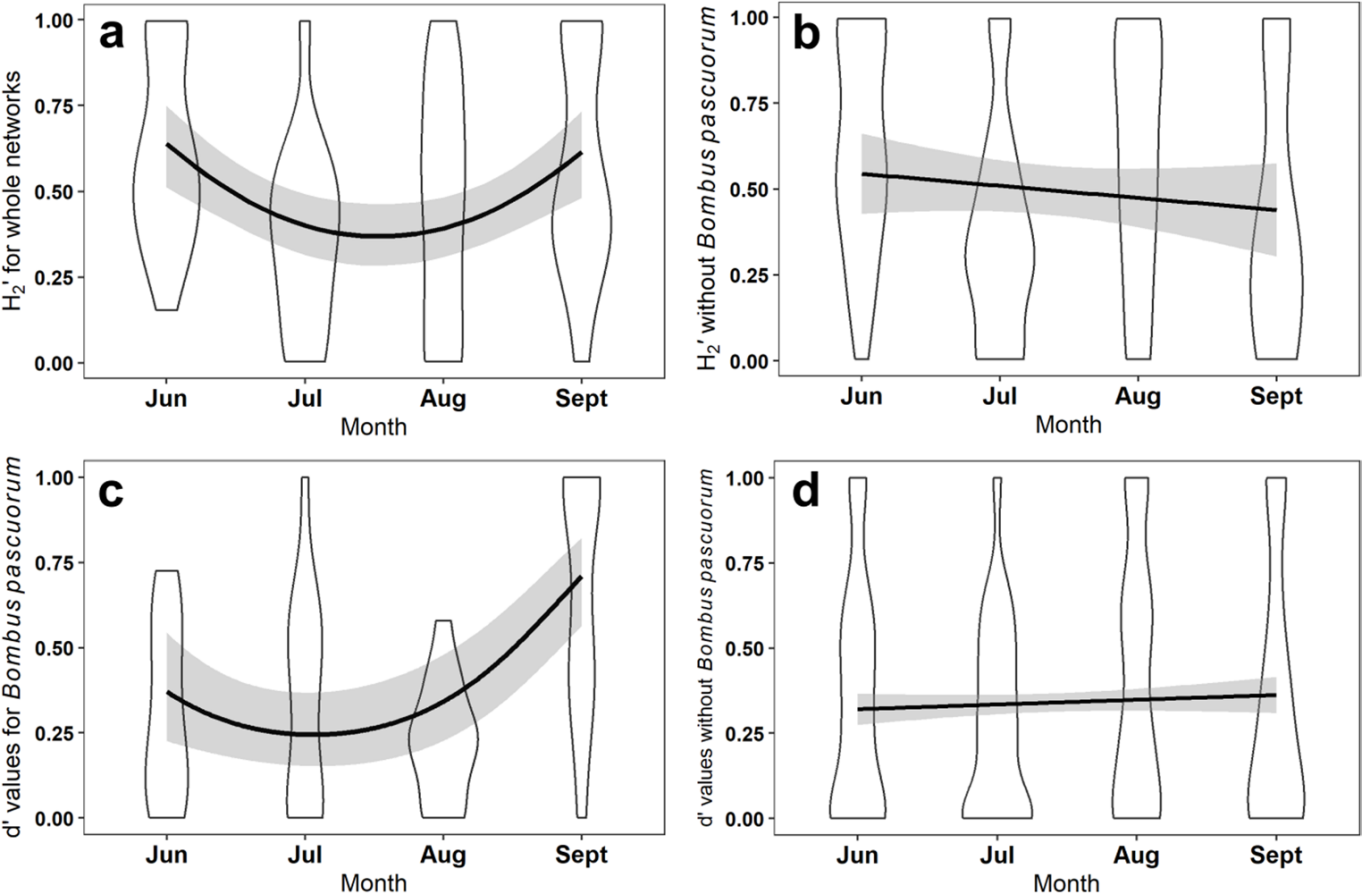
**a**) H_2_’ when calculated for whole networks including Bombus pascuorum, showed variation across the months of the study, increasing in June and September and decreasing in July and August. **b**) H_2_’ when calculated without B.pascuorum interactions did not show any variation across the four months of the study. **c**) species specialisation in *B.pascuorum* showed variation between months, decreasing in July and August and increasing in September. **d**) Flower visitor specialisation (d’) when *B.pascuorum* was filtered from the data set showed no variation across the four months of the study. Violin plots show the spread of network indices calculated in each month for each garden and duplicated across native and non-native plants. Lines in graphs a & c show predicted values from the model fit and lines in b & d show the lines of best fit to the raw data. Grey shading represents the 95% confidence intervals

Final models for network structure, with and without *B. pascuorum* can be seen in Table 1. All indices of network structure (H_2_’, Interaction evenness and WNODF) were influenced by month (H_2_’: Estimate = 2.55, z = 3.86, *p* < 0.001; Fig. 5a, Interaction evenness: Estimate= −0.40, z=−2.642, *p* = 0.019; WNODF: Estimate= −22.67, z=−2.09, *p* = 0.038). No other explanatory variables influenced network structure. Similarly, month was the only predictor variable of flower visitor specialisation (d’: Estimate = 0.61, z = 2.162, *p* = 0.031).

When removing *B. pascuorum* from networks, network specialisation (H_2_’) did not vary across the four months of the study (Fig. 5b), nor were they influenced by any of the variables tested. However, when analysing the specialisation of *B. pascuorum* only, month had a strong influence (Estimate = 2.94, z = 4.19 *p* < 0.001), with specialisation increasing in September (Fig. 5c). Yet species specialisation values for all species excluding *B. pascuorum* showed no differences across the four months (Fig. 5d).

## Discussion

Private residential gardens contribute to a large proportion of land within a city. Small enhancements to floral plantings, such as increasing floral abundance and richness may help to increase pollinator diversity, as our study found greater floral richness and abundances were associated with more flower visits. Importantly, our results suggest that such measures could be effective in any garden, regardless of its position within the urban matrix. Finally, plant-flower visitor network structure varied seasonally which appeared to be driven largely by the increasing abundance of a dominant species and its interactions with native and non-native plants. We discuss the implications for garden management below.

### Native and non-native plants increase flower visits

Our study showed the importance of native plants within private gardens, as at the community level, native flowers were associated with more floral visits than non-native flowers. Other studies have found that increasing floral resources within a green space can increase flower visits, and that native plants receive more flower visits (Chrobock et al. 2013; Zaninotto et al. 2023). Despite this, non-native plants still had a role in flower visitor networks in our study. We found that increasing the abundance of non-natives was linked to a higher number of flower visits. Our results are consistent with other studies where certain non-native plant species received a high proportion of flower visits (Kovács-Hostyánszki et al. 2022; Salisbury et al. 2015), as 10% of flower visits were to *Lavandula*, a non-native plant. Furthermore, at the plant species level, non-native plant species received a higher richness of floral visitors than native plants. However, this was not observed when assessing the richness of floral visitors in the whole community. This result further supports that certain non-native species are attractive to pollinators, as the difference in flower visitor richness was only evident at the plant species level. In our study, it is likely that non-natives had a role in pollinator networks due to the large proportion of generalist flower visitors; for example, *B. pascuorum* accounted for 25% of all floral visits. Generalist pollinators can forage on a range of plant species; thus, increasing the abundance of non-native plants may provide additional foraging opportunities for pollinators which can reduce competition and allow more generalist species to forage on these resources. However, whether increased floral abundance alleviates competition for floral resources also depends on resources quality (pollen and nectar nutritional content), which was not measured in this study.

Interestingly, the percentage of plant species visited by flower visitors within networks was higher for non-native than native plants. Previously, it has been found that certain native plants, such as *Bellis perennis* and *Myosotis arvensis*, can be unsuitable for pollinators, in that they provide little nectar and pollen resources (Hicks et al. 2016). Moreover, plant origin may not be the only variable to influence flower visits, plant morphology (flower shape and colour), as well as life cycles (annual or perennial), may play important roles in attracting flower visitors (Lowenstein et al. 2019).

Overall, regardless of plant origin and the surrounding landscape, an increase in either richness or abundance was associated with greater flower visitor abundance and richness, respectively. Many studies have reported the positive associations of floral diversity with pollinator richness and abundance (Baldock et al. 2019; Fowler et al. 2016; Salisbury et al. 2015; Seitz et al. 2020; Simao et al. 2018; Theodorou et al. 2017), with one study showing how small-scale interventions, such as sowing “mini meadows”, can boost pollinator diversity (Griffiths-Lee et al. 2022).

### Seasonal changes in non-native plant abundance

Floral richness and abundance of non-native plants increased from June until September, whilst a decreasing trend was observed in native plants. Previous studies have found non-native plants with later flowering times become more dominant later in the season (Zaninotto et al. 2023), receive similar or more flower visits (Salisbury et al. 2015; Seitz et al. 2020; Staab et al. 2020; Zaninotto et al. 2023), and substitute the role of native plants (Staab et al. 2020). Thus, it has been argued that the later blooming period of non-native plants can be used to provide floral resources across a longer time period (Staab et al. 2020). In our study, floral visits to native and non-native plants did not match changes in the seasonal abundance of these plants. Furthermore, we found a large variation in native plant abundance across gardens in September, showing some gardens can provided high native floral resources later in the season. We argue that it is unnecessary to supplement gardens with large numbers of non-native plants later in the season. This is particularly important given that some invasive non-natives can harm ecosystem functioning (Aizen et al. 2008).

### Seasonal changes in network structure

We highlighted seasonal variations in network structure where nestedness and interaction evenness were lower in June and September; the opposite trend was seen for network specialisation. However, these seasonal patterns disappeared when networks were calculated whilst excluding *B. pascuorum* visits. Furthermore, when *B. pascuorum* was removed from data, pollinator specialisation did not vary across the season, further supporting its role as the main driver of these dynamics. While seasonal changes in network structure are often attributed to an influx of generalist pollinators later in the season (Luder et al. 2018) or temporal changes in resource availability (Souza et al. 2018), our results suggest that a single dominant species can determine network structure. Experimental studies have shown that the removal or addition of dominant species can influence network structure (Brosi et al. 2017; Worthy et al. 2023). For example, Worthy et al’s study (2023) highlight how increased dominance of honeybees in plant-pollinator networks can change network structure. This change in network structure occurred through the addition of honeybee interactions rather than by altering wild bee interactions with plants. However, it is important to note that in our study, the removal of *B.pascuorum* from networks was simulated and networks excluding this species were still observed in the field where *B.pascuorum* was present.

Moreover, closer inspection of *B.pascuorum’s* foraging behaviour showed a marked increase in visits to non-native plants in September, which coincided with higher specialisation values for *B. pascuorum*. Several mechanisms have been proposed to drive short term specialisation of generalist pollinators. Firstly, optimal diet theory suggests that abundant plant species can result in pollinator specialisation due to the cost effectiveness of foraging on these plants (Suni et al., 2022). A high floral abundance of plant species in urban areas has been shown to influence the short-term specialisation of pollinators, as urban pollinators carry more conspecific pollen than rural pollinators despite similar pollinator diversity (Suni et al., 2022). Secondly, interspecific competition can drive specialisation through niche partitioning. Studies have found that *B. pascuorum* is a central, generalist species yet, may reconfigure its interactions with plants in the presence of high floral diversity (Fisogni et al. 2022). Furthermore, as the most abundant pollinator in the community, *B. pascuorum* may displace other species from floral resources (Wignall et al. 2020), thereby reinforcing its own interactions with a narrower subset of plants. Alternatively, this pattern may reflect phenological matching (Glaum et al. 2021), since the non-native species it visited most frequently were abundant in September.

Network structure is thought to be strongly related to the robustness of a network (Proesmans et al. 2024; Theodorou et al. 2017). Increasing link density increases network robustness (Proesmans et al. 2024 ), whilst nestedness buffers networks against extinction cascades (Proesmans et al. 2024). Therefore, the seasonal changes to network structure found here may translate to more vulnerable networks in June and September. Thus, we suggest that a higher abundance of floral resources should be provided for pollinators at the end of the season, to buffer against negative effects on network structure.

### Management recommendations and conservation context

To support pollinator diversity in gardens, we recommend maintaining high floral richness of native plants throughout the season. Although our study found that non-native floral abundance increased visitation more than native abundance, this effect was not evident for floral richness. Moreover, several non-native species were highly preferred by pollinators, but the invasive potential of non-native plants makes them unsuitable for broad recommendation. Instead, we suggest prioritizing native species that attract diverse pollinators, including later-flowering taxa such as *Geranium sp.*, *Allium sp.*, and *Fragaria sp*. While we did not compare gardens to other urban green spaces, previous work shows that private gardens collectively cover large areas, exceeding other habitats in supporting pollinator diversity at the city scale (Baldock et al. 2019). Our findings complement these studies by demonstrating how increasing floral richness within gardens can enhance the contribution of gardens across a city to urban pollinator conservation.

## Limitations

Our study contributes to understanding plant–flower visitor interactions in urban green spaces, particularly private residential gardens, where research is limited. Although restricted to one city and year, we believe our findings are transferable to other European cities with extensive green spaces, such as Glasgow. The absence of an effect of surrounding green space on flower visits may be more pronounced in cities with little green cover.

Sampling was constrained to June–September and to 20-minute observation periods per flower. Short observation times can bias results toward the most abundant species, but our approach was relatively long compared to similar timed studies (e.g., Sirohi et al. 2022). Furthermore, within the study period, we observed clear monthly differences in network structure, suggesting greater seasonal variation had spring been included. Recruitment through volunteer participation may also have introduced bias, though our gardens ranged from pollinator-friendly habitats to intensively managed lawns, capturing useful variation.

Finally, to identify visitors to species level, individuals were removed from the system. While this may alter interactions by removing cues or reducing subsequent visits, it is standard in network studies and ensures our results remain comparable with others (Baldock et al. 2019; Herrmann et al. 2023; Sirohi et al. 2022; Staab et al. 2020; Zaninotto et al. 2023).

## Conclusions

Our study shows that increasing floral richness in gardens is a key driver of pollinator diversity. Importantly, this effect is maintained regardless of where gardens are located within the urban landscape. Both native and non-native plants can contribute to floral richness, with some non-native plants being particularly attractive to several pollinator species. However, the predominance of generalist species in our study suggests that more native plants may be required to support specialist pollinators across the season. Overall, given that private gardens make up around 30% of urban green space in Great Britain (Office for National Statistics (ONS), 2023), even small increases in floral richness could enhance pollinator diversity at the city scale.

## Supplementary information

Below is the link to the electronic supplementary material.

ESM 1 (DOCX 2.96 MB)

## Data Availability

Data is available at [https://doi.org/10.5525/gla.researchdata.1962] (https://doi.org/10.5525/gla.researchdata.1962).
