# Supplementary material for: Supporting pollinators in urban gardens: floral richness and abundance influence flower visitor interactions regardless of the surrounding landscape
Source: Urban Ecosyst. 2025 Nov 14;28(6):235. doi: 10.1007/s11252-025-01848-7 (PMC12618392; doi:10.1007/s11252-025-01848-7)
Supplement: Supplementary file 1 — (DOCX 2.96 MB) [file 11252_2025_1848_MOESM1_ESM.docx]

# Online resource

Contents

[S.1- Sample site locations 1](#_Toc210394718)

[S.2- Flower visitor species 2](#_Toc210394719)

[S.3- Plant species 4](#_Toc210394720)

[S.4- Flower visitor plant preferences 9](#_Toc210394721)

[S.5- Optimal models 12](#_Toc210394722)

[S.6- Flower visits at the plant level 15](#_Toc210394723)

## S.1- Sample site locations


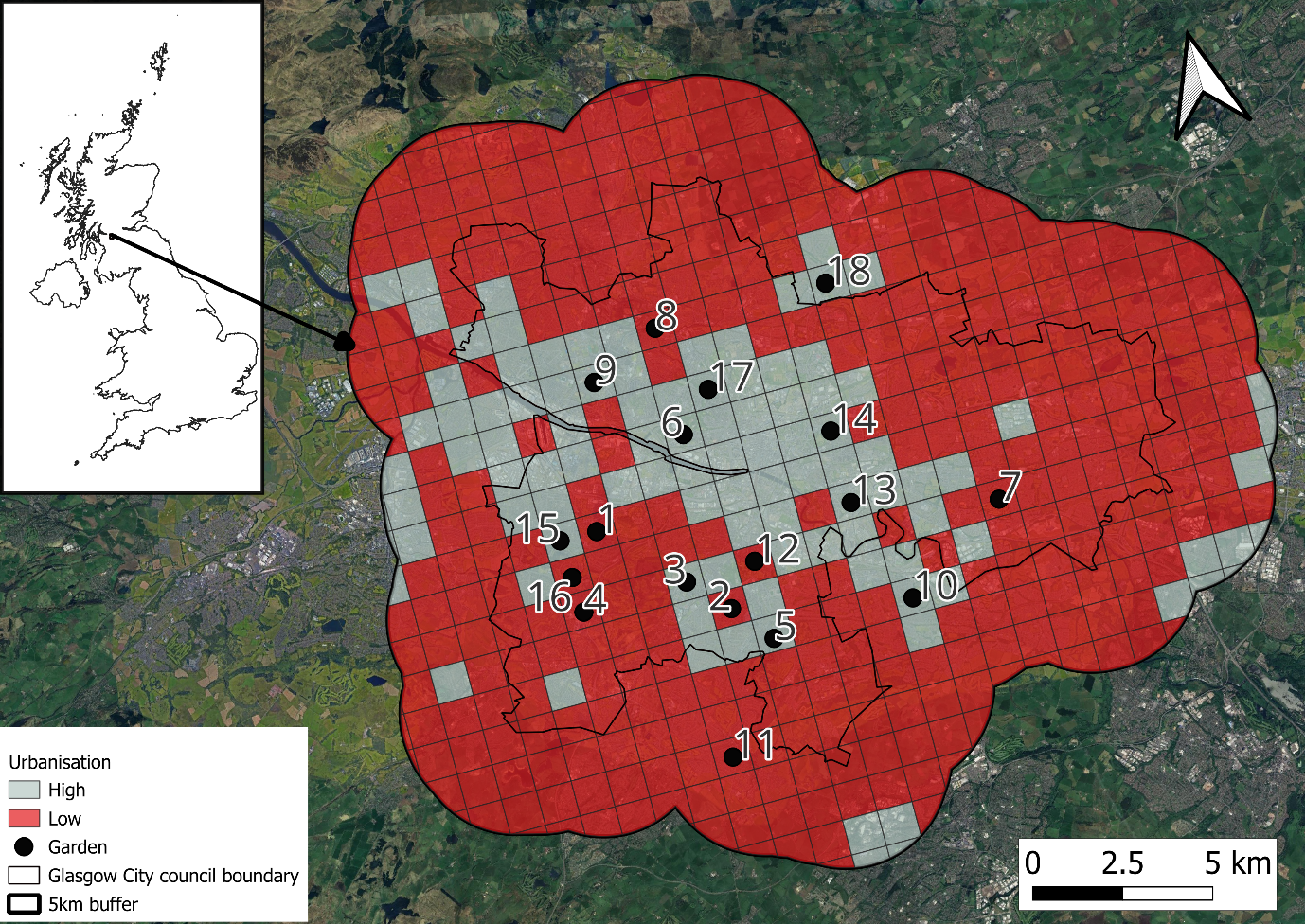


**Figure S 1:** A map showing the locations of private gardens sampled in the study. Gardens were located across Glasgow City Council including a 5km buffer of the council boundary. Gardens were at least 1km apart from each other and stratified across high(grey) and low (red) urbanisation categories.

## S.2- Flower visitor species

| Flower visitor | Abundance |
| --- | --- |
| *Bombus pascuorum* | 256 |
| *Bombus lucorum.agg./ terrestris* | 174 |
| *Episyrphus balteatus* | 82 |
| *Apis mellifera* | 73 |
| *Bombus hypnorum* | 47 |
| *Platycheirus albimanus* | 46 |
| *Bombus pratorum* | 37 |
| *Bombus lapidarius* | 35 |
| *Eristalis tenax* | 23 |
| *Syrphus ribesii* | 23 |
| *Syritta pipiens* | 22 |
| *Eupeodes corollae* | 19 |
| *Melanostoma scalare* | 18 |
| *Bombus hortorum* | 16 |
| *Megachilidae centuncularis* | 11 |
| *Colletes daviesanus* | 9 |
| *Eristalis pertinax* | 8 |
| *Helophilus pendulus* | 8 |
| *Syrphus torvus* | 8 |
| *Syrphus vitripennis* | 8 |
| *Lasioglossum smeathmanellum* | 7 |
| *Osmia bicornis* | 7 |
| *Meliscaeva cinctella* | 7 |
| *Eupeodes luniger* | 6 |
| *Lasioglossun fratellum* | 5 |
| *Syrphus spp.* | 5 |
| *Meredon equestris* | 4 |
| *Myathropa florea* | 4 |
| *Lasioglossum albipes* | 3 |
| *Lasioglossum leucopus* | 3 |
| *Sphegina verecunda* | 3 |
| *Syrphus rectus/vitripennis* | 3 |
| *Pieris rapae* | 3 |
| *Lasioglossum calceatum* | 2 |
| *Baccha elongata* | 2 |
| *Chrysogaster solstitialis* | 2 |
| *Dasysryphus albostriatus* | 2 |
| *Dasysrphus tricinctus* | 2 |
| *Platycheirus scutatus* | 2 |
| *Sphaerophoria scripta* | 2 |
| *Pieris brassicae* | 2 |
| *Vanessa atalanta* | 2 |
| *Andrena subopaca* | 1 |
| *Anthophora furcata* | 1 |
| *Hylaeus hyalinatus* | 1 |
| *Megachilidae willughbiella* | 1 |
| *Sphecodes geoffrellus* | 1 |
| *Cheilosia bergenstammi* | 1 |
| *Epistrophe grossulariae* | 1 |
| *Eristalis horticola* | 1 |
| *Meligramma guttatum* | 1 |
| *Meliscaeva auricollis* | 1 |
| *Melanostoma mellinum* | 1 |
| *Xylota segnis* | 1 |
| *Aglais urticae* | 1 |

**Table S 1**: A list of flower visitor species recorded in private gardens from June until September. The table shows the total abundance of each flower visitor across the study. Bombus lucorum agg. and Bombus terrestris were grouped together. Furthermore, certain Syrphus species were unable to be identified to species, thus were named at the genus level.

## S.3- Plant species

| Plants | Plant origin | Floral Abundance |
| --- | --- | --- |
| *Bellis perennis* | Native | 1202 |
| *Geranium* | Native | 407 |
| *Lavandula* | Non native | 386 |
| *Brassica rapa* | Native | 280 |
| *Lobelia* | Native | 184 |
| *Salvia* | Native | 164 |
| *Epilobium* | Native | 161 |
| *Hydrangea* | Non native | 130 |
| *Aster* | Non native | 129 |
| *Ranunculus* | Native | 125 |
| *Rhinanthus* | Native | 118 |
| *Trifolium repens* | Native | 116 |
| *Cymbalaria murialis* | Non native | 112 |
| *Jacobaea vulgaris* | Native | 101 |
| *Fallopia baldschuanica* | Non native | 97 |
| *Jasminum* | Non native | 96 |
| *Buddleja* | Non native | 95 |
| *Alchemilla mollis* | Native | 92 |
| *Mentha* | Native | 92 |
| *Pelargonium* | Non native | 89 |
| *Phlox* | Non native | 82 |
| *Crocosmia* | Non native | 77 |
| *Origanum vulgare* | Native | 75 |
| *Eupatorium* | Native | 72 |
| *Lysimachia vulgaris* | Native | 72 |
| *Eruca vesicaria* | Non native | 71 |
| *Ligustrum ovalifolium* | Native | 71 |
| *Papaver* | Native | 71 |
| *Digitalis purpurea* | Native | 69 |
| *Cotoneaster* | Native | 65 |
| *Campanula* | Native | 64 |
| *Hieracium* | Native | 63 |
| *Petunia* | Non native | 61 |
| *Rosa* | Native | 59 |
| *Philadelpheus* | Non native | 55 |
| *Begonia* | Non native | 51 |
| *Hypericum* | Native | 49 |
| *Spiraea* | Non native | 48 |
| *Myosotis arvensis* | Native | 43 |
| *Prunella vulgaris* | Native | 42 |
| *Ocimum basilicum* | Non native | 39 |
| *Borago officinalis* | Non native | 38 |
| *Pilosella aurantiaca* | Native | 38 |
| *Erica* | Native | 36 |
| *Veronica* | Native | 35 |
| *Fragaria* | Native | 32 |
| *Geum urbanum* | Native | 32 |
| *Lupinus* | Non native | 32 |
| *Leucanthemum vulgare* | Native | 31 |
| *Clematis* | Native | 30 |
| *Linaria vulgaris* | Native | 30 |
| *Eryngium* | Native | 27 |
| *Impatiens walleriana* | Native | 27 |
| *Lobularia* | Non native | 27 |
| *Primula* | Native | 27 |
| *Raphanus* | Native | 26 |
| *Silene dioica* | Native | 25 |
| *Malva* | Native | 24 |
| *Auberita* | Non native | 23 |
| *Erysimum* | Non native | 23 |
| *Fuschia* | Non native | 23 |
| *Centaurea cyanus* | Native | 22 |
| *Matthiola* | Native | 22 |
| *Sambucus* | Native | 22 |
| *Sedum* | Native | 22 |
| *Lotus corniculatus* | Native | 21 |
| *Petrosedum* | Native | 21 |
| *Solanum lycopersicum* | Native | 21 |
| *Allium* | Native | 20 |
| *Astilbe* | Non native | 20 |
| *Rhododendron* | Non native | 19 |
| *Bistorta* | Non native | 18 |
| *Foeniculum vulgare* | Non native | 18 |
| *Symphyotrichum* | Non native | 18 |
| *Tropaeolum majus* | Non native | 17 |
| *Oenothera biennis* | Non native | 16 |
| *Oxalis acetosella* | Native | 16 |
| *Rubus* | Native | 16 |
| *Silene coronaria* | Native | 16 |
| *Centaurea nigra* | Native | 15 |
| *Prunella* | Native | 15 |
| *Achillea millefolium* | Native | 14 |
| *Antirrhinum majus* | Non native | 14 |
| *Scabiosa* | Native | 13 |
| *Delphinium* | Non native | 12 |
| *Echinops* | Non native | 12 |
| *Alcea rosea* | Non native | 11 |
| *Calendula officinalis* | Non native | 11 |
| *Phedimus* | Non native | 11 |
| *Libertia grandiflora* | Non native | 10 |
| *Thymus vulgaris* | Native | 10 |
| *Aquilegia* | Native | 9 |
| *Chamaenerion* | Native | 9 |
| *Daucus carota* | Native | 9 |
| *Sonchus* | Native | 9 |
| *Viola tricolor* | Native | 9 |
| *Chamaemelum* | Native | 8 |
| *Hedera* | Native | 8 |
| *Iberis* | Native | 8 |
| *Anemone* | Native | 7 |
| *Arabis* | Non native | 7 |
| *Taraxacum agg.* | Native | 7 |
| *Celosia argentea* | Non native | 6 |
| *Helianthus annuus* | Non native | 6 |
| *Solidago* | Native | 6 |
| *Tagets patula* | Non native | 6 |
| *Hylotelephium* | Unknown | 5 |
| *Lythrum salicaria* | Native | 5 |
| *Phacelia tanacetifolia* | Non native | 5 |
| *Stellaria* | Native | 5 |
| *Cyclamen* | Non native | 4 |
| *Leycesteria formosa* | Non native | 4 |
| *Cephalaria gigantea* | Non native | 3 |
| *Fagopyrum* | Non native | 3 |
| *Lonicera* | Native | 3 |
| *Orchis* | Native | 3 |
| *Trifolium pratense* | Native | 3 |
| *Viburnum* | Native | 3 |
| *Alstroemeria* | Non native | 2 |
| *Armeria maritima* | Native | 2 |
| *Lapsana communis* | Native | 2 |
| *Nepeta* | Native | 2 |
| *Betonica officinalis* | Unknown | 1 |
| *Calystegia sepium* | Native | 1 |
| *Cucurbita pepo* | Non native | 1 |
| *Delosperma* | Non native | 1 |
| *Geum rivale* | Native | 1 |

**Table S 2**: A list of plants observed within gardens from June until September. The table shows the total floral abundance recorded for each plant as well as its origin status for Great Britain (Native or Non-native). Plants were identified to the lowest taxonomic level (genus or species).

## S.4- Flower visitor plant preferences

| Plants | Flower visitor | Observed | Null | SES | Native_NonNative | Month |
| --- | --- | --- | --- | --- | --- | --- |
| *Allium* | *B..pascuorum* | 2 | 0.29 | 3.431 | Native | September |
| *Anemone* | *S..ribesii* | 3 | 0.1 | 8.700 | Native | September |
| *Bistorta* | *B..pascuorum* | 7 | 1.38 | 4.427 | Non native | September |
| *Chamaenerion* | *B..pascuorum* | 4 | 0.11 | 11.272 | Native | September |
| *Fragaria* | *B..pascuorum* | 2 | 0.03 | 11.490 | Native | September |
| *Geranium* | *E..balteatus* | 4 | 0.84 | 3.669 | Native | September |
| *Lavandula* | *B..pascuorum* | 24 | 3.92 | 10.231 | Non native | September |
| *Papaver* | *B..lucorum.agg.* | 2 | 0.05 | 8.902 | Native | September |
| *Papaver* | *E..balteatus* | 2 | 0.14 | 4.357 | Native | September |
| *Pelargonium* | *C..solstitialis* | 2 | 0.14 | 4.939 | Non native | September |
| *Fallopia baldschuanica* | *E..pertinax* | 2 | 0.25 | 3.500 | Non native | September |
| *Fallopia baldschuanica* | *Syrphus spp.* | 2 | 0.09 | 6.641 | Non native | September |
| *Fallopia baldschuanica* | *A..mellifera* | 5 | 0.3 | 8.141 | Non native | September |
| *Scabiosa* | *B..pascuorum* | 3 | 0.1 | 8.700 | Native | September |
| *Sonchus* | *S..ribesii* | 3 | 0.07 | 11.426 | Native | September |
| *Symphyotrichum* | *E..tenax* | 4 | 0.5 | 5.080 | Non native | September |
| *Symphyotrichum* | *B..pascuorum* | 6 | 1.98 | 3.224 | Non native | September |
| *Allium* | *B..pascuorum* | 3 | 0.56 | 3.634 | Native | August |
| *Bistorta* | *B..pascuorum* | 2 | 0.28 | 3.482 | Non native | August |
| *Bistorta* | *A..mellifera* | 2 | 0.09 | 5.953 | Non native | August |
| *Buddleja* | *A..mellifera* | 2 | 0.12 | 5.756 | Non native | August |
| *Chamaenerion* | *A..mellifera* | 6 | 0.11 | 18.730 | Native | August |
| *Chamaenerion* | *B..lucorum.agg* | 3 | 0.17 | 7.015 | Native | August |
| *Echinops* | *S..vitripennis* | 2 | 0 | NA | Non native | August |
| *Echinops* | *B..pascuorum* | 2 | 0.08 | 6.244 | Non native | August |
| *Echinops* | *A..mellifera* | 2 | 0.04 | 9.952 | Non native | August |
| *Fuschia* | *B..pascuorum* | 3 | 0.54 | 3.247 | Non native | August |
| *Geranium* | *M..scalare* | 3 | 0.4 | 4.197 | Native | August |
| *Lavandula* | *B..lapidarius* | 5 | 0.88 | 4.356 | Non native | August |
| *Lavandula* | *B..pascuorum* | 15 | 7.43 | 2.900 | Non native | August |
| *Lavandula* | *B..lucorum.agg* | 17 | 2.67 | 8.841 | Non native | August |
| *Matthiola* | *B..pascuorum* | 2 | 0.15 | 5.155 | Native | August |
| *Mentha* | *S..pipiens* | 3 | 0.36 | 4.715 | Native | August |
| *Mentha* | *P..albimanus* | 9 | 0.46 | 14.000 | Native | August |
| *Reynoutria* | *M..cinctella* | 2 | 0.26 | 3.591 | Non native | August |
| *Reynoutria* | *S..verecunda* | 2 | 0.08 | 7.042 | Non native | August |
| *Scabiosa* | *B..pascuorum* | 2 | 0.03 | 11.490 | Native | August |
| *Solidago* | *C..daviesanus* | 4 | 0 | NA | Native | August |
| *Solidago* | *S..pipiens* | 2 | 0.05 | 8.902 | Native | August |
| *Aqueliga* | *B..lucorum.agg* | 2 | 0.31 | 3.477 | Native | July |
| *Aqueliga* | *B..hypnorum* | 2 | 0.22 | 4.046 | Native | July |
| *Campanula* | *B..lapidarius* | 2 | 0.08 | 7.042 | Native | July |
| *Cotoneaster* | *B..hypnorum* | 4 | 1.33 | 2.444 | Native | July |
| *Delphinium* | *B..lucorum.agg* | 2 | 0.31 | 3.213 | Non native | July |
| *Epilobium* | *P..albimanus* | 2 | 0.12 | 5.278 | Native | July |
| *Epilobium* | *E..corollae* | 2 | 0.2 | 4.221 | Native | July |
| *Geranium* | *B..pascuorum* | 8 | 3.09 | 2.641 | Native | July |
| *Geranium* | *M..equestris* | 3 | 0.31 | 5.312 | Native | July |
| *Hieracium* | *B..lapidarius* | 2 | 0.04 | 9.952 | Native | July |
| *Hypericum* | *B..hypnorum* | 6 | 0.24 | 11.641 | Native | July |
| *Papaver* | *B..lucorum.agg* | 9 | 1.53 | 5.859 | Native | July |
| *Papaver* | *B..hypnorum* | 4 | 0.69 | 4.425 | Native | July |
| *Ranunculus* | *B..pratorum* | 9 | 0.81 | 8.174 | Native | July |
| *Rhododendron* | *B..pratorum* | 3 | 0.31 | 4.377 | Non native | July |
| *Rhododendron* | *O..bicornis* | 2 | 0.07 | 7.526 | Non native | July |
| *Rhododendron* | *B..hypnorum* | 4 | 0.34 | 6.829 | Non native | July |
| *Rubus* | *B..hypnorum* | 4 | 0.2 | 9.452 | Native | July |
| *Rubus* | *B..pratorum* | 4 | 0.16 | 9.724 | Native | July |
| *Salvia* | *B..pascuorum* | 4 | 0.24 | 7.933 | Native | July |
| *Sambucus* | *B..hypnorum* | 3 | 0.43 | 3.287 | Native | July |
| *Taraxacum* | *B..lucorum.agg* | 2 | 0.19 | 4.319 | Native | July |
| *Aqueliga* | *B..hypnorum* | 2 | 0.11 | 5.477 | Native | June |
| *Campanula* | *B..lapidarius* | 2 | 0.02 | 14.072 | Native | June |
| *Epilobium* | *P..albimanus* | 2 | 0.17 | 4.279 | Native | June |
| *Epilobium* | *E..corollae* | 2 | 0.22 | 3.850 | Native | June |
| *Geranium* | *B..pascuorum* | 8 | 2.81 | 3.270 | Native | June |
| *Geranium* | *M..equestris* | 3 | 0.26 | 4.878 | Native | June |
| *Hieracium* | *B..lapidarius* | 2 | 0.03 | 11.490 | Native | June |
| *Hypericum* | *B..hypnorum* | 6 | 0.27 | 12.236 | Native | June |
| *Lupinus* | *B..lucorum.agg* | 4 | 0.96 | 2.810 | Non native | June |
| *Papaver* | *B..lucorum.agg* | 9 | 1.12 | 7.201 | Native | June |
| *Papaver* | *B..hypnorum* | 4 | 0.7 | 4.011 | Native | June |
| *Ranunculus* | *B..pratorum* | 9 | 1.3 | 7.144 | Native | June |
| *Rhododendron* | *B..pratorum* | 3 | 0.35 | 4.033 | Non native | June |
| *Rhododendron* | *O..bicornis* | 2 | 0.04 | 9.952 | Non native | June |
| *Rhododendron* | *B..hypnorum* | 4 | 0.4 | 5.811 | Non native | June |
| *Rubus* | *B..hypnorum* | 4 | 0.23 | 8.914 | Native | June |
| *Rubus* | *B..pratorum* | 4 | 0.17 | 9.494 | Native | June |
| *Salvia* | *B..lucorum.agg* | 2 | 0.37 | 2.996 | Native | June |
| *Salvia* | *B..pascuorum* | 4 | 0.34 | 6.600 | Native | June |
| *Sambucus* | *B..hypnorum* | 3 | 0.5 | 3.354 | Native | June |
| *Taraxacum* | *B..lucorum.agg* | 2 | 0.19 | 4.319 | Native | June |

**Table S 3:** Results from the null model analysis in the econullnetr package, showing the plants species that certain flower visitor species interacted with more than expected based on chance whilst accounting for the floral abundance of each plant. Here networks from each garden were grouped together but separated by month. The observed value shows the number of flower visitors observed visiting the plant, and the expected value is the number of flower visits expected to visit the plant based on its floral abundance. SES is the standard error values with the plant origin (Native or Non-native) and month of the observation shown in the table.

## S.5- Optimal models

| **Response** | **Predictors** | **DF** | ***X*^2^** | **P value** |
| --- | --- | --- | --- | --- |
| **Floral visits** | | | | |
| Flower visits at the  community | Floral richness  Origin  Month  Floral abundance/ m^2^  Floral abundance*Origin | 1  1  1  1  1 | 14.32  5.00  8.47  3.94  4.80 | <0.001  0.035  0.004  0.047  0.041 |
| Flower visitor richness at the community | Floral richness  Floral abundance * Origin | 1  1 | 33.97  4.16 | <0.001  0.041 |
| Flower visits at the plant level | Month  Floral abundance | 1  1 | 10.84  11.32 | 0.002  0.001 |
| Flower visitor richness at the plant level | Month  Origin  Floral abundance | 1  1  1 | 10.03  3.91  11.20 | 0.002  0.048  0.001 |
| Percentage of plants to receive visits | Origin | 1 | 5.58 | 0.027 |
| *B. pascuourm* flower visits | Floral richness  Floral abundance  Origin  Month*Origin  Floral richness * Origin | 1  1  1  1  1 | 9.44  46.62  6.00  52.28  16.56 | 0.002  <0.001  0.027  <0.001  <0.001 |
| Flower visits excluding *B.pascuorum* | Floral abundance  Floral richness  Month | 1  1  1 | 10.98  29.42  13.52 | 0.001  <0.001  <0.001 |
| Flower visitor richness excluding *B.pascuorum* | Floral abundance  Floral richness  Month  Origin | 1  1  1  1 | 8.96  60.89  6.23  4.66 | 0.003  <0.001  0.013  0.036 |
| **Floral resource** | | | | |
| Floral abundance | Month  Month^2  Origin  Month*Origin | 1  1  1  1 | 14.46  5.82  6.97  13.85 | <0.001  0.024  0.027  <0.001 |
| Floral richness | Origin  Month*Origin | 1  1 | 44.44  10.09 | <0.001  0.001 |
| **Network indices** | | | | |
| H2’ | Month^2 | 1 | 14.46 | <0.001 |
| Interaction eveness | Month^2 | 1 | 6.72 | 0.019 |
| WNODF | Month^2 | 1 | 4.31 | 0.038 |
| D’ pollinators | Month^2 | 1 | 4.98 | 0.031 |
| D’ for *B.pascuorum* | Month^2 | 1 | 16.74 | <0.001 |
| **Network indices without *B.pascuorum*** | | | | |
| H2’ | NULL |  |  |  |
| Interaction evenness | NULL |  |  |  |
| WNODF | NULL |  |  |  |
| D' pollinators | NULL |  |  |  |

**Table S 4:** The table shows the explanatory variables selected to explain floral visitation (the number and richness of flower visitors at the community level, the number and richness of flower visitors at the plant species level and the percentage of plants that were interacted with in the community), floral abundance and floral richness in gardens and network structure (H_2_, NODF, Interaction evenness and d’ for pollinators). Variables were selected by a backwards step wise selection process. The degrees of freedom (df), chi squared (x^2^) values and p values for which the variables were selected on are displayed in the table. P values shown in the table were adjusted using the FDR method.
